# Ceragenins in Combination with Ivacaftor Prevent the Formation of Biofilm by Bacteria That Cause Rhinosinusitis

**DOI:** 10.3390/pharmaceutics18010001

**Published:** 2025-12-19

**Authors:** Szczepan Kaliniak, Piotr Deptuła, Jakub Spałek, Mariusz Sawieljew, Sylwia Chmielewska-Deptuła, Tamara Daniluk, Agata Lesiak, Bonita Durnaś, Paul B. Savage, Ewelina Piktel, Robert Bucki, Sławomir Okła

**Affiliations:** 1 Department of Medical Microbiology and Nanobiomedical Engineering, Medical University of Białystok, 15-222 Białystok, Poland; szczepan.kaliniak@onkol.kielce.pl (S.K.); mariusz.sawieljew@umb.edu.pl (M.S.); sylwia.chmielewska@umb.edu.pl (S.C.-D.); tamara.daniluk@umb.edu.pl (T.D.); buckirobert@gmail.com (R.B.); 2 Department of Otolaryngology, Head and Neck Surgery, Holy-Cross Cancer Center, Artwińskiego 3, 26-734 Kielce, Poland; jakub.spalek@ujk.edu.pl (J.S.); bonita.durnas@onkol.kielce.pl (B.D.); 3 Independent Laboratory of Nanomedicine, Medical University of Białystok, 15-222 Białystok, Poland; piotr.deptula@umb.edu.pl (P.D.); ewelina.piktel@umb.edu.pl (E.P.); 4 Institute of Medical Science, Collegium Medicum, Jan Kochanowski University of Kielce, IX Wieków Kielce 19A, 25-317 Kielce, Poland; agatalesiak@wp.pl; 5 Department of Chemistry and Biochemistry, Brigham Young University, Provo, UT 84602, USA; paul_savage@byu.edu

**Keywords:** rhinosinusitis, sinusitis, biofilm, bacterial biofilm, ceragenins, colistin, vancomycin, meropenem, ivacaftor

## Abstract

**Background/Objectives:** Ceragenins (CSAs) maintain strong antibacterial activity even in cystic fibrosis (CF) sputum. Ivacaftor (IVA), a CF transmembrane regulator modulator, provides significant clinical benefits in CF therapy. Based on these properties, we hypothesized that the combination of CSAs and IVA, due to their antibacterial and biofilm-penetrating abilities, may also be beneficial in the treatment of chronic rhinosinusitis (CRS), including CRS in CF patients. Notably, the physicochemical properties of biofilms in chronic rhinosinusitis (CRS) resemble those in CF sputum. **Methods:** We determined the minimal inhibitory and bactericidal concentrations (MIC and MBC) and the fractional inhibitory concentration index (FICI) for ceragenins (CSA-13, CSA-44, CSA-131), ivacaftor (IVA), selected conventional antibiotics, and their combinations against both reference and clinical strains. Bacterial viability within biofilms was also evaluated following exposure to these agents. Atomic force microscopy (AFM) was used to analyze the morphology and nanomechanical properties of *Staphylococcus aureus* and *Pseudomonas aeruginosa*. In addition, rheological measurements of *Pseudomonas aeruginosa* biofilms treated with CSAs combined with IVA were performed using a rotational rheometer. **Results:** The tested agents demonstrated anti-biofilm activity against bacterial strains associated with CRS development. IVA enhanced the anti-biofilm effects of both CSAs and tested antibiotics. CSAs exhibited low MIC and MBC values, confirming their efficacy against tested pathogens. AFM showed that CSA-44, IVA, vancomycin, and their combinations altered the nanomechanical properties of *Pseudomonas aeruginosa* and *Staphylococcus aureus* cells. Interestingly, the addition of IVA induced aggregation of *S. aureus* cells. CSAs reduced the stiffness of *P. aeruginosa* biofilms, and co-treatment with IVA resulted in a further decrease in biofilm stiffness. **Conclusions:** These findings indicate that ceragenins, particularly in combination with ivacaftor, represent a promising therapeutic strategy for challenging chronic infections caused by the studied bacteria. This supports further research aimed at developing new treatment methods for CRS.

## 1. Introduction

Rhinosinusitis (RS) is a prevalent inflammatory condition affecting the nasal and paranasal sinus mucosa. The European Position Paper on Rhinosinusitis and Nasal Polyps 2020 categorizes RS into acute rhinosinusitis (ARS) and chronic rhinosinusitis (CRS) based on the duration and types of symptoms. ARS is characterized by symptoms lasting less than or equal to 12 weeks, while CRS has symptoms persisting for equal to or more than 12 weeks and is distinguished into primary and secondary types. Symptoms like nasal blockage, facial pain, cough, and loss of smell are common indicators of RS. This classification further considers anatomical distribution (localized vs. diffused), endotype dominance (type 2 inflammation vs. non-type 2 inflammation), and clinical phenotype. ARS affects around 6–15% of the general population and is a leading cause of antibiotic prescriptions. On the other hand, CRS is a significant health concern globally, impacting about 5–12% of the worldwide population, including approximately 10.9% of the population in Europe and 11.9% in the US [1,2,3]. Moreover, the overall indirect cost related to CRS-related losses in work productivity is estimated to be in excess of USD 20 billion per year in the US [4]. CRS, its symptoms and consequences, significantly reduces the quality of life in affected patients by causing nasal obstruction and smell disorders, reducing sleep quality, and even affecting mental health [5]. RS is caused mainly by viruses (rhinoviruses, adenovirus, influenza virus, parainfluenza virus). On the other hand, etiological factors of CRS are biofilm-forming bacteria and, less frequently, fungi. Examples of frequent bacteria that cause CRS are *S. aureus*, *Streptococcus pneumoniae*, *P. aeruginosa*, *Haemophilus influenzae*, and *Enterobacterales* rods. Furthermore, fungal infections caused by, e.g., Candida or *Aspergillus* may occur in immunocompromised patients [6,7,8,9,10,11,12].

Bacterial biofilms are strongly associated with CRS and play key roles in the pathogenesis of CRS [13]. A biofilm is a complex aggregation of microbial cells that are encased in a self-produced extracellular polymeric substance (matrix) that can form on both biotic and abiotic surfaces. This type of growth enhances bacterial survival by providing protection against environmental stresses, antibiotics, and the immune system. Thus, biofilms contribute to the chronicity and resilience of bacterial infections [14,15,16]. Considering the critical role of biofilm in the development of chronic sinusitis, it is important to research, develop, and apply biofilm-targeted treatments. Anti-biofilm strategies include physical removal of biofilm, disruption or removal of the biofilm matrix, use of chemical agents like antibiotics and antimicrobial peptides, disruption of cellular communication, and efforts to eliminate subpopulations of dormant cells. These activities are aimed at eliminating biofilm and improving penetration of anti-biofilm agents through the biofilm matrix. New anti-biofilm strategies include the use of nanotechnology-based materials [17], disruption of extracellular deoxyribonucleic acid (DNA) [18], natural compounds, quorum sensing inhibition, enzymatic degradation of the biofilm matrix, proteins, lipopolysaccharides, exopolysaccharides, and secondary messengers involved in various signaling pathways [19,20,21,22]. Overall, in biofilm-targeted therapy, many compounds remain in the development phase, including metallic nanoparticles [23], bacteriophages [24], S-nitrosoglutathione [25], monoterpenes [26], manuka honey [27], and ciprofloxacin and azithromycin sinus stents [28].

The use of ceragenins to disrupt established biofilms as an alternative to standard antibiotic therapy is a promising approach [29,30]. Ceragenins are synthetic antimicrobial agents designed to mimic the physicochemical properties of natural antimicrobial peptides. They are derivatives of cholic acid that have been modified to carry cationic charges, allowing them to disrupt microbial membranes and effectively kill a broad range of pathogens, including drug-resistant bacteria and fungi [31,32,33]. Ceragenins exhibit activity against both planktonic and biofilm-associated forms of microorganisms, including bacterial strains resistant to standard antibiotics, such as colistin-resistant *Klebsiella pneumoniae* and methicillin-resistant *S. aureus* [34]. Another advantage of ceragenins in comparison with antimicrobial peptides is that they maintain their properties regardless of changes in their environment, such as high salt concentrations or the presence of negatively charged biopolymers [35]. Ceragenins CSA-13, CSA-44, CSA-131, CSA-192, and CSA-255 display antimicrobial activity against *Candida* spp., *Streptococcus*, *Enterococcus,* and *Bacteroides* spp. found in the oral cavity [30,36,37]. CSA-13, CSA-44, and CSA-131 are also promising alternatives in the eradication of *P. aeruginosa* and *S. aureus.* Ceragenin CSA-131 has high activity against clinical bacterial strains of *P. aeruginosa* and is characterized by anti-biofilm activity, minimal likelihood of developing resistant organisms, lower toxicity, and better stability in blood compared to standard antibiotics [32,38]. CSA-44 and CSA-131 also exhibit activity against strains of bacteria resistant to chlorhexidine and colistin [39]. Furthermore, CSA-13 and CSA-131 demonstrate activity against anaerobic bacteria, which may also be involved in chronic sinusitis [40].

CRS is also an important clinical problem in patients with cystic fibrosis (CF) and is responsible for disease progression. CF transmembrane conductance regulator potentiators, including ivacaftor (IVA), which enhances chloride secretion in respiratory epithelia, can be used successfully against microbial biofilms. Furthermore, a previous report suggests that apart from its standard activity justifying use in the treatment of CF, IVA shows a weak inhibitory effect on bacterial DNA gyrase and topoisomerase IV. Moreover, IVA enhances the antimicrobial activity of ciprofloxacin against *P. aeruginosa* [41,42]. And IVA may enhance the anti-biofilm effect of other substances, such as L-methionine; co-treatment of IVA and L-methionine can downgrade the formation of *P. aeruginosa* biofilm [43].

Here we provide experimental data, including AFM observation, indicating the potential of ceragenins used alone and in combination with IVA in developing new strategies to treat patients with CRS.

## 2. Materials and Methods

### 2.1. Bacteria Strains and Synthesis of Ceragenins

The following reference strains of bacteria were used: *Staphylococcus aureus* (ATCC 29213), *Streptococcus pneumoniae* (ATCC 49619), *Moraxella catarrhalis* (ATCC 25238), *M. catarrhalis* (ATCC 496), *Haemophilus influenzae* (ATCC 49766), *Pseudomonas aeruginosa* (ATCC 27853), and *P. aeruginosa* (ATCC 27863). All indicated bacterial strains were cultured under aerobic conditions at a temperature of 35–37 degrees Celsius for a minimum of 24 h on Columbia Agar with Sheep Blood (Oxoid, Hampshire, UK) and Haemophilus Selective Agar (Oxoid) for *H. influenzae.* Changes in bacterial metabolic activity upon addition of tested molecules were monitored using *P. aeruginosa* Xen5 and *S. aureus* MRSA Xen30 strains engineered through conjugation and transposition of a plasmid carrying transposon Tn5 luxCDABE (purchased from Caliper Life Science Inc., Mountain View, CA, USA). All clinical strains were isolated from microbiological samples taken from patients with clinical signs of upper respiratory tract infection and sent to the laboratory for routine microbiological diagnostics. The identification of the bacterial strains studied was performed using MALDI-TOF MS mass spectrometry, utilizing the MALDI Biotyper system from Bruker (Billerica, MA, USA). This system identifies microorganisms based on their protein profile, comparing it to an extensive, constantly updated database. This study was approved by the Ethical Committee for Human Studies of the Jan Kochanowski University in Kielce, nr 11/2025, and the guidelines outlined in the Declaration of Helsinki were followed. Samples were cultured according to the relevant laboratory procedures. The following clinical strains were tested: *Staphylococcus aureus* MRSA, *Streptococcus pneumoniae*, *Streptococcus intermedius*, *Streptococcus anginosus*, *Streptococcus pyogenes*, *Pseudomonas aeruginosa*, and *Pseudomonas putida*. The ceragenins were synthetized following previously described procedures [44].

### 2.2. Evaluation of MIC and MBC

Minimal inhibitory concentrations (MICs) and minimal bactericidal concentrations (MBCs) were determined for ceragenins CSA-13, CSA-44, and CSA-131, ivacaftor (IVA), vancomycin (VAN), colistin (COL), and meropenem (MEM) using broth microdilution assays performed in accordance with the guidelines of EUCAST [45]. Specifically, serial 2-fold dilutions in 96-well microtiter plates (from 256 µg/mL to 0.5 µg/mL) of the tested ceragenins were prepared in Mueller-Hinton Broth (for *S. aureus* and *Pseudomonas*) or brain heart infusion (BHI) broth (for *Streptococcus*, *Hemophilus*, and *Moraxella*). The appropriate inoculum of each isolate was prepared by collecting colonies from fresh 18–24 h culture grown on solid medium (Muller-Hinton agar for *S. aureus* and *Pseudomonas*, Muller-Hinton supplemented with 5% blood + 20 mg/L NAD for *Streptococcus*, *Hemophilus*, and *Moraxella*) and resuspending them in broth medium prior to adding them to the wells to a final concentration of 5 × 10^5^ CFU/mL per well. The incubation was performed at 35 ± 1 °C for 18 ± 2 h under aerobic conditions. MIC values were read macroscopically as the lowest concentrations of the antimicrobial agent that inhibited microbial growth. MICs for ceragenins and relevant antibiotics were considered as additional controls (vancomycin for Gram-positive bacteria, meropenem for *M. catarrhalis* and *H. influenzae*, colistin for Gram-negative—*P. aeruginosa*) were determined both for reference and clinical strains. After determination of MICs, MBC values were determined by planting samples from wells on agar plates (dedicated to specific strains—as above) and incubating at 37 °C under aerobic conditions for an additional 18 ± 2 h. MBC values were read as the concentrations at which no bacterial growth was observed on the solid medium.

### 2.3. Evaluation of FICI

Fractional inhibitory concentration indices (FICIs) were determined to evaluate the synergistic effect of ceragenins (CSA-13, CSA-44, and CSA-131) with conventional antibiotics (tested in a 1:1 ratio). To assess FICI, the following formula was used: FICa = MIC (ab)/MIC (a), FICb = MIC (ab)/MIC (b), and FICI = FICa + FICb (where MICa and MICb are MIC values of compounds used separately, and MICab-MIC are values of compounds used in combination) [46,47].

### 2.4. Chemiluminescence Intensity

Chemiluminescence intensity measurement was performed to evaluate the effect of tested molecules on the metabolic activity of *P. aeruginosa* Xen 5 and *S. aureus* MRSA Xen 30 strains (bioluminescent strains derived from the parental strains *P. aeruginosa* ATCC 19660 and clinical strain *S. aureus* I6, respectively). Due to the introduction of a luminescent operon into *Pseudomonas* and *Staphylococcus* strains, it is possible to monitor the effects of an antibacterial compound in real time. As a result, these tests are highly sensitive, provide quick readings, and allow the effects to be detected after only a short period of exposure, which can be difficult to observe using traditional culture methods. To perform this, bacteria were grown to the mid-log phase at 37 °C on the appropriate medium, then resuspended in Luria–Bertani broth and adjusted to 10^8^ CFU/mL. In a subsequent step, *P. aeruginosa* Xen 5 and *S. aureus* MRSA Xen 30 were exposed to CSA-13, CSA-44, CSA-131, and IVA, at concentrations of 5, 10, 20, and 40 μg/mL, and their activity was compared with colistin (for *P. aeruginosa* Xen 5) or vancomycin (for *S. aureus* Xen 30), which were considered as additional controls. Changes in bacterial chemiluminescence during 30 min after a compound addition were recorded using a microplate reader (Varioskan LUX, Thermo Fisher Scientific, Waltham, MA, USA). Statistical analysis was determined using a one-way ANOVA test.

### 2.5. Evaluation of Biofilm Formation

Biofilm formation of *S. aureus* (ATCC 29213), *P. aeruginosa* (ATCC 27853), and *M. catarrhalis* (ATCC 496) upon 24 h exposure to ceragenins CSA-13, CSA-44, CSA-131, and COL, VAN, and MEM (at doses ranging from 0.1 to 20 µg/mL) was explored using resazurin-based fluorometric staining to visualize live cells within biofilm mass in microtiter plates [48]. Tested agents were examined alone and when combined with IVA (at a concentration of 5 µg/mL). Biofilm formation in treated samples was normalized to untreated control (0 µg/mL; 100% viability).

### 2.6. AFM Study

The impact of ceragenins and antibiotics alone and in combination with ivacaftor on the morphology and nanomechanical properties of the *S. aureus* (ATCC 29213) and *P. aeruginosa* (ATCC 27853) cells was determined using a NanoWizard 4 BioScience atomic force microscope (AFM; JPK/Bruker, Billerica, MA, USA) operating in Quantitative Imaging (QI) mode (JPK QI™ mode). *S. aureus* (ATCC 29213) and *P. aeruginosa* (ATCC 27853) planktonic cells were resuspended in PBS (OD_600_: ~0.1), followed by incubation with CSA-44 (10 µg/mL), VAN (1 µg/mL), and COL (1 µg/mL) and in combination with 5 µg/mL of IVA at 37 °C for 1 h. Then, 100 µL aliquots of bacterial samples were transferred onto a mica surface previously functionalized with 0.01% Poly-L-Lysine. QI maps from control and treated cells were acquired using Bruker MSCT-A AFM (Billerica, MA, USA) probes featuring a nominal spring constant of 0.07 N/m. Topography maps of 20 × 20 µm and 3 × 3 µm were recorded with a resolution of 128 pixels per line under wet conditions. Small maps (3 × 3 µm) were also used to determine the physicochemical properties of the microorganisms. QI Height Mode was applied to visualize the topography; QI Slope Mode to assess surface stiffness; QI Adhesion Mode to measure adhesion forces between the AFM probe and cells.

### 2.7. Biofilm Rheology

An assessment of the impact of ceragenins CSA-13 and CSA-13 combined with IVA on the rheological properties of *P. aeruginosa* (ATCC 27853) biofilms was conducted using a strain-controlled Anton Paar MCR702e rheometer (Anton Paar GmbH, Graz, Austria) equipped with parallel-plate geometry (upper plate diameter—25 mm). A mature, 3-day-old biofilm was examined. Biofilm was prepared using Petri dishes with 15 mL of LB broth where 100 µL of inoculum (OD600 = 0.1) prepared from a fresh 24 h culture on LB agar was added. The bacteria were incubated at 37 °C for 72 h. After incubation, the medium was decanted and the biofilm remaining on the dish was collected using a cell scraper into a Falcon tube. A 500 µL of the collected biofilm was transferred onto the lower plate of the rheometer [49]. The compounds were introduced following biofilm maturation and applied directly to the prepared sample on the rheometer. This approach prevented disruption of the biofilm architecture that could occur during post-incubation transfer to the lower rheometer plate. Throughout the incubation period, the biofilm was maintained under controlled temperature and humidity conditions to minimize evaporative loss. The rheological testing protocol consisted of an oscillating shear strain test with a frequency f = 1 Hz and an amplitude γ = 1% for 60 s. The course of the storage modulus (G’) of biofilms over time was determined.

### 2.8. Assessment of Biocompatibility of Ceragenins in Combination with Ivacaftor

To evaluate the potential cytotoxic effects of the tested compounds—ceragenins, ivacaftor, and their combination—three assays were performed: (i) hemolysis assay, (ii) MTT assay, and (iii) investigation of cellular morphology upon addition of tested formulations.

To explore hemocompatibility of tested compounds, blood collected from healthy donors was centrifuged (2500 rpm, 4 °C, 10 min) and erythrocytes from the pellet were resuspended in sterile PBS to obtain a hematocrit of approx. 5%. Red blood cell suspension was then exposed to increasing concentrations of the tested agents for 24 h at 37 °C. Upon centrifugation of samples, supernatants were collected and transferred to clear 96-well plates to measure absorbance at 590 nm. RBCs with sterile PBS and 0.1% Triton X-100 were used as negative and positive controls, respectively.

Simultaneously, the viability of fibroblast cells derived from mouse embryonic tissue (NIH Swiss 3T3 line, maintained in DMEM supplemented with 10% fetal bovine serum [FBS] and 1% antibiotics) treated with these compounds was investigated using the MTT assay. To do so, NIH/3T3 cells were seeded in 96-well plates at a density of 1 × 10^4^/well and exposed to increasing concentrations of tested agents for 24 h at 37 °C. After incubations, cells were washed twice with sterile PBS, and MTT working solution (1 mg/mL in PBS) was added for 4 h. Next, the working solution was removed, cells were washed with PBS, and insoluble formazan was dissolved in 100 µL of sterile DMSO before absorbance measurement at 540 nm. Untreated cells with cell culture medium only were considered as a negative control (100% viability).

Images for geometric analysis were acquired using a Leica DMi8 optical microscope (10× objective, Wetzlar, Germany) equipped with an environmental chamber. The cells were cultured and transferred into a 96-well plate in DMEM medium (ATCC, Manassas, VA, USA) supplemented with 10% FBS. The compounds (CSA-13 and IVA) were added immediately before initiating the time-lapse imaging sequence. Additionally, to assess the potential side effects of DMSO (used as a solvent for IVA, additional control), cells were also exposed to 0.2% DMSO—a concentration corresponding to that present in the ivacaftor-treated samples. Cells were imaged for up to 4 h at 10 min intervals. Pictures from the 4 h time period were used for analysis. For each treatment condition, images from four independent wells were analyzed. Image processing and measurements were performed using ImageJ software (version 1.53h). The following morphometric parameters were determined: cell area, circularity, and aspect ratio.

### 2.9. Statistical Analysis

Results of chemiluminescence analysis and rheology study are presented as mean ± SE, while results of biofilm viability assays, AFM study, and biocompatibility experiments are demonstrated as mean ± SD. The significance of differences was determined using one-way ANOVA with Tukey’s test. Results with a *p*-value under 0.05 were considered statistically significant.

## 3. Results

### 3.1. Antibacterial Activity of Ceragenins, Ivacaftor, and Antibiotics Against Tested Reference and Clinical Strains

Among all tested ceragenins, CSA-13 demonstrated the most potent activity, with MIC values below 0.5 μg/mL across most tested strains (Table 1). For *S. aureus* (including reference strain ATCC 29213 and MRSA clinical isolates), the MIC was 0.5 μg/mL, with MBC reaching 2 μg/mL. Low MIC (<0.5 µg/mL) and MBC (2 µg/mL) were determined for *S. aureus* (ATCC 29213), which underscores the potent activity of CSA-13 against this microorganism. Similar high efficacy of tested agents was observed for *S. pneumoniae* (MICs below 0.5 μg/mL) and *M. catarrhalis* (MIC = 0.5 μg/mL) strains. CSA-44 and CSA-131 also displayed substantial activity against tested microorganisms, generally with slightly higher MIC and MBC values compared to CSA-13.

IVA overall showed high MICs (often ≥1 μg/mL and up to 128 μg/mL for some strains). With *S. aureus*, IVA exhibited varying levels of effectiveness. MRSA strains had MIC values ranging from 2 to 8 µg/mL. When testing *S. pneumoniae* (ATCC 49619), IVA showed an MIC of 1 µg/mL which is relatively low compared to other bacteria in this group. Clinical isolates of *S. pneumoniae* have MIC values ranging from 0.5 to 8 µg/mL, indicating moderate efficacy. For *H. influenzae* (ATCC 49766), IVA demonstrated an MIC below 0.5 µg/mL, highlighting potential high effectiveness against this pathogen. In contrast, *M. catarrhalis* (ATCC 25238) had an MIC of 0.5 µg/mL, suggesting moderate susceptibility to IVA. In the case of *P. aeruginosa*, IVA showed relatively weak activity, with an MIC exceeding 128 µg/mL even for the ATCC 27853 strain. Similarly, clinical isolates of *P. aeruginosa* exhibited high MIC values, requiring concentrations as high as 256 µg/mL and above, underscoring its limited efficacy against this pathogen.

Ranges of MIC values for VAN were from <0.5 to 1 µg/mL in all tested clinical strains of *S. aureus*, which displayed susceptibility to this drug according to the EUCAST break points [50]. All tested clinical strains of *S. pneumoniae* were susceptible to vancomycin (MIC values from <0.5 to 1 µg/mL) as well as *S. anginosus* and *S. intermedius.* Similarly, all tested strains of G-negative bacilli showed susceptibility to COL and MEM, respectively.

### 3.2. FICI Evaluation

FICIs with *P. aeruginosa* (ATCC 27853) demonstrated synergy with CSA-13 (0.265) and COL (0.128) with IVA (Table 2). Significant synergy was also noted with the CSA-13/COL combination (0.1875) for *P. aeruginosa* (ATCC 27853). The other combinations generally displayed indifference.

### 3.3. Luminescence-Based Analysis

**Figure 1:**
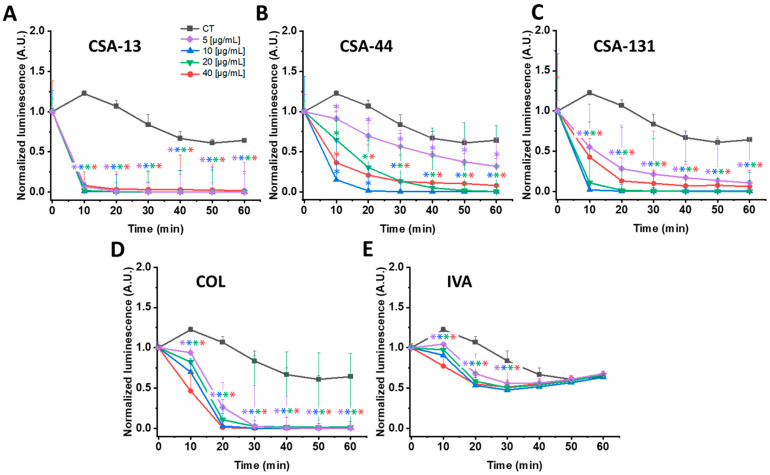
Changes in *P. aeruginosa* Xen 5 luminescence upon addition of CSA-13 (**A**), CSA-44 (**B**), CSA-131 (**C**), colistin (COL) (**D**), and ivacaftor (IVA) (**E**) tested at concentrations of 5, 10, 20, and 40 μg/mL in comparison to untreated control. Data represent mean ± SE, *n* = 3. * indicates statistical significance compared to untreated bacteria cells (*p* ≤ 0.05).

**Figure 2:**
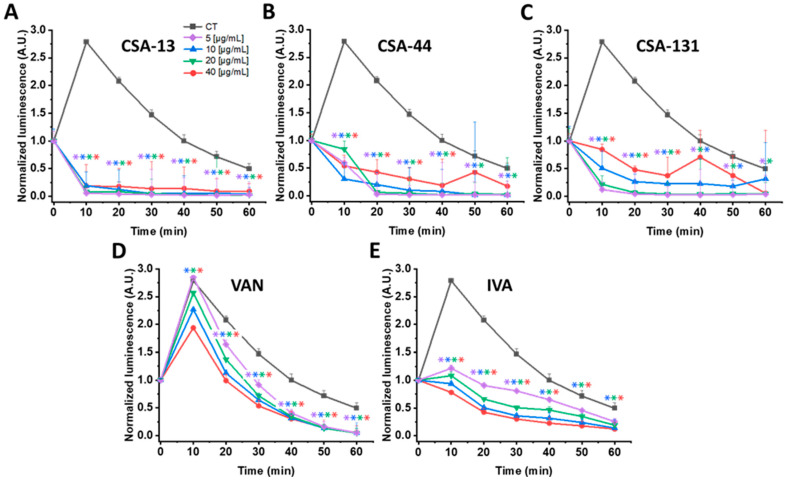
Changes in *S. aureus* Xen 30 luminescence upon addition of CSA-13 (**A**), CSA-44 (**B**), CSA-131 (**C**), vancomycin (VAN) (**D**), and ivacaftor (IVA) (**E**) tested at concentrations of 5, 10, 20, and 40 μg/mL in comparison to untreated control. Data represent mean ± SE, *n* = 3. * indicates statistical significance compared to untreated bacteria cells (*p* ≤ 0.05).

*P. aeruginosa* Xen 5 and *S. aureus* MRSA Xen 30 luminescence was recorded following the addition of CSA-13, CSA-44, CSA-131, and IVA, and antibiotics such as COL and VAN, and is presented in Figure 1 and Figure 2. Measurement of luminescence served as a proxy for bacterial metabolic activity, enabling rapid assessment of growth and viability.

As shown in Figure 1, the majority of the tested compounds significantly reduced the luminescence of *P. aeruginosa* Xen 5. Among them, CSA-13 (Figure 1A) demonstrated the most potent decrease, suggesting an antimicrobial effect, inducing a 93–99% decrease in chemiluminescence within 10 min and reaching 97–99% after 60 min. For the remaining ceragenins, luminescence decreased by 26–87% for CSA-44 and 65–92% for CSA-131 after 10 min, respectively (Figure 1B,C). Exposure to conventional antibiotics such as COL resulted in a reduction in luminescence ranging from 23 to 62% within 10 min (Figure 1D). Notably, IVA exhibited only a minor effect, causing a 14–37% decrease in luminescence after 10 min and a negligible 1–2% reduction after 60 min (Figure 1E). These findings suggest that IVA alone has a minimal impact on *P. aeruginosa* Xen 5 metabolic activity, supporting its potential use in combination therapy.

Regarding *S. aureus* Xen 30, CSA-13 also produced a statistically significant reduction in bacterial metabolic activity and potential viability (Figure 2). A decrease of 93–98% was observed after 10 min and 82–96% after 60 min (Figure 2A). Treatment with CSA-44 and CSA-131 resulted in substantial luminescence decreases of 69–89% and 69–95% after 10 min, respectively (Figure 2B,C). Conversely, treatment with conventional antibiotic VAN resulted in a maximum decrease of 32% after 10 min at a concentration of 40 µg/mL, underscoring its comparatively weaker impact on *S. aureus* Xen 30 metabolism and survival (Figure 2D). IVA, however, reduced luminescence by 57–72% after 10 min and by 49–76% after 60 min (Figure 2E). Interestingly, IVA exhibited stronger antibacterial activity against *S. aureus* Xen 30 than against *P. aeruginosa* Xen 5. Therefore, these findings collectively justify its application in combination with ceragenins to enhance antibacterial efficacy.

### 3.4. S. aureus (ATCC 29213), P. aeruginosa (ATCC 27853), and M. catarrhalis (ATCC 4961) Biofilm Formation in the Presence of Tested Agents

**Figure 3:**
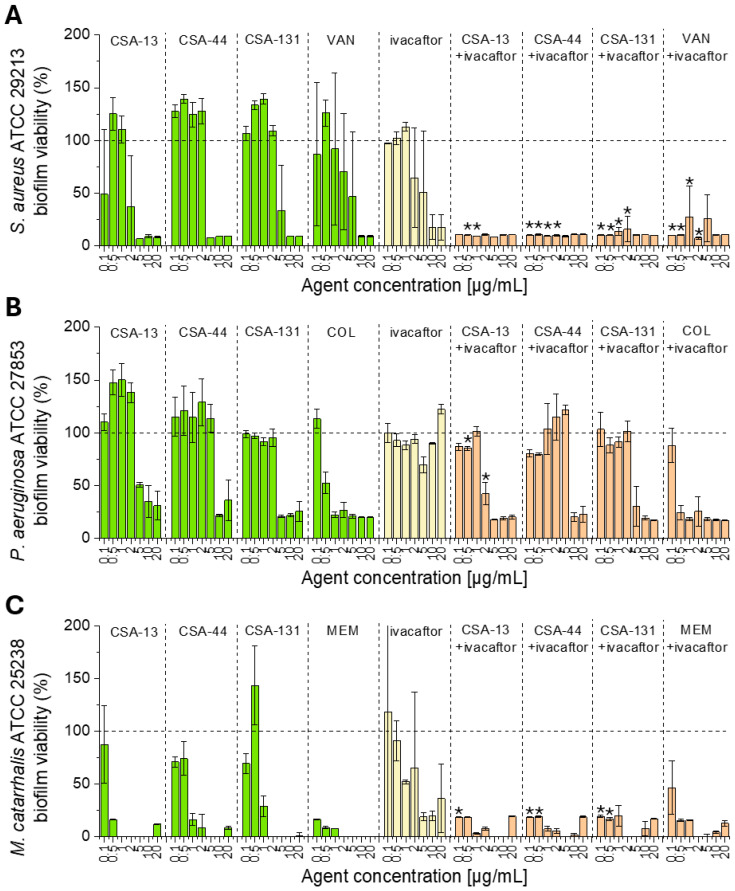
The formation of *S. aureus* (ATCC 29213) (**A**), *P. aeruginosa* (ATCC 27853) (**B**), and *M. catarrhalis* (ATCC 25238) (**C**) biofilm in the presence of ceragenins CSA-13, CSA-44, CSA-131, vancomycin (VAN), colistin (COL), and meropenem (MEM) alone (green bars), in the presence of ivacaftor (yellow bars) (all at doses ranging from 0.1 to 20 µg/mL) and when combined with IVA (5 µg/mL) (orange bars). Biofilm formation in treated samples was normalized to the untreated control (0 µg/mL; 100% viability; indicated as black horizontal line). Results are presented as a mean ± SD from 3 technical replicates. * indicates statistical significance (*p* < 0.05) when compared to single treatments.

CSA-13, CSA-44, and CSA-131 exhibit antibacterial activity against *S. aureus* (ATCC 29213), *P. aeruginosa* (ATCC 27853), and *M. catarrhalis* (ATCC 49616), reducing biofilm formation in a concentration-dependent manner (Figure 3). Effects were most noticeable at the highest concentrations (10 and 20 µg/mL). The effectiveness of ceragenins depends on the bacterial species. Ceragenin activity can be enhanced when combined with conventional antibiotics or IVA. Such combination led to stronger inhibition of biofilm growth compared to the use of a single agent.

### 3.5. AFM Assessment of the Topography and Nanomechanical Properties of S. aureus and P. aeruginosa Cells After Treatment with the Tested Agents

AFM was employed to visualize structural and nanomechanical alterations of bacterial cell surfaces of *S. aureus* and *P. aeruginosa* upon exposure to the tested compounds. Representative images of bacteria treated with ceragenin CSA-44, either alone or in combination with IVA, as well as with antibiotics—VAN for Gram-positive *S. aureus* and COL for Gram-negative *P. aeruginosa*—administered individually and in combination with IVA, are presented.

**Figure 4:**
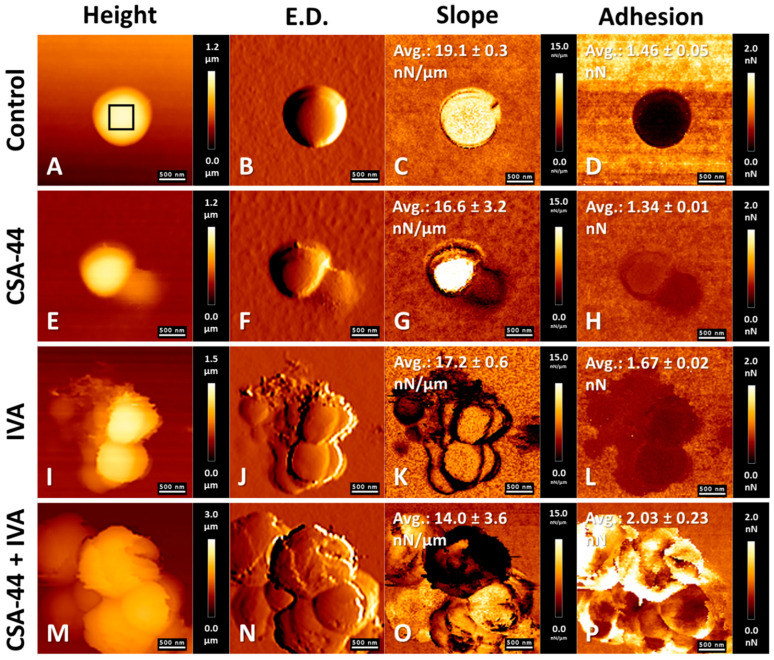
Effects of CSA-44 (10 µg/mL) and ivacaftor (IVA) (5 µg/mL) on *S. aureus* cells. Atomic force microscopy (AFM) was used to assess topographical and nanomechanical properties under various modes: Height (**A**,**E**,**I**,**M**), Edge Detection (**B**,**F**,**J**,**N**), Slope (**C**,**G**,**K**,**O**), and adhesion (**D**,**H**,**L**,**P**). Panels (**A**–**D**) represent untreated control cells; panels (**E**–**H**) correspond to cells exposed to CSA-44; panels (**I**–**L**) show cells treated with IVA; and panels (**M**–**P**) illustrate the effects of combined treatment with CSA-44 and IVA. A representative region used for nanomechanical parameter calculations is marked within panel (**A**). The calculated mean values ± SD are displayed within the respective panels. For each experimental condition, the values of the nanomechanical parameters were calculated from five separate bacteria cells.

Figure 4 shows AFM results for *S. aureus* cells. Figure 4A–D represent the control, untreated cells. Surface topography (Figure 4E,F) and nanomechanical property maps (Figure 4G,H) confirmed the antibacterial activity of CSA-44, as evidenced by morphological alterations. Mechanical properties analysis demonstrated reduced stiffness and adhesion in comparison with control cells. Figure 4I–L illustrate the effects of IVA, which resulted in increased adhesion (Figure 4L) and enhanced cellular aggregation (Figure 4I,J). The combination of CSA-44 and IVA induced pronounced aggregation of *S. aureus* (Figure 4M,N), accompanied by surface alterations and nanomechanical changes, including decreased stiffness (Figure 4O) and increased adhesion (Figure 4P). The stiffness of bacteria treated with CSA-44 decreased by 13%, and those treated with IVA by nearly 10%. The combination of CSA-44 and IVA resulted in a reduction in cell stiffness of more than 26%. Regarding cell surface adhesion, CSA-44 induced a slight decrease, whereas IVA treatment increased adhesion by 14% relative to untreated bacteria. In contrast, the combined treatment with CSA-44 and IVA led to a 39% increase in adhesiveness.

**Figure 5:**
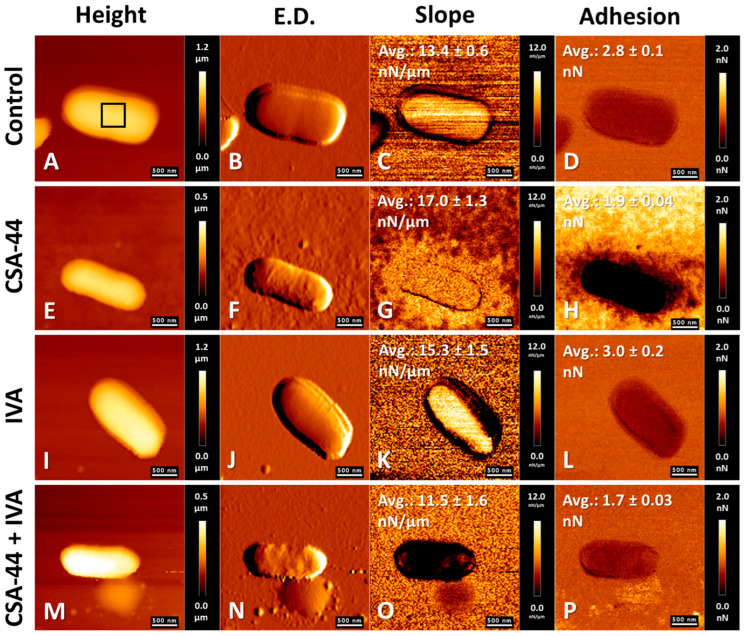
Effects of CSA-44 (10 µg/mL) and ivacaftor (IVA) (5 µg/mL) on *P. aeruginosa* cells. Atomic force microscopy (AFM) was used to assess topographical and nanomechanical properties under various modes: Height (**A**,**E**,**I**,**M**), Edge Detection (**B**,**F**,**J**,**N**), Slope (**C**,**G**,**K**,**O**), and adhesion (**D**,**H**,**L**,**P**). Panels (**A**–**D**) represent untreated control cells; panels (**E**–**H**) correspond to cells exposed to CSA-44; panels (**I**–**L**) show cells treated with IVA; and panels (**M**–**P**) illustrate the effects of combined treatment with CSA-44 and IVA. A representative region used for nanomechanical parameter calculations is marked within panel (**A**). The calculated mean values ± SD are displayed within the respective panels. For each experimental condition, the values of the nanomechanical parameters were calculated from five separate bacteria cells.

AFM analyses for *P. aeruginosa* are displayed in Figure 5. CSA-44 induced morphological alterations, with surface changes evident in Figure 5F compared with untreated cells (Figure 5A–D). In contrast to *S. aureus*, no structural alterations or aggregation were observed following ivacaftor treatment (Figure 5J). Nevertheless, adhesion increased, consistent with the effect observed in *S. aureus*. The combination of CSA-44 and IVA led to alterations in morphology and mechanical properties, including a reduction in cell height (also observed with CSA-44 alone) and frequent leakage events (Figure 5N), indicative of cell wall and membrane disruption. In this case, treatment with CSA-44 or IVA alone led to increases in stiffness of nearly 27% and 14%, respectively. In contrast, the combined application of these compounds resulted in a 14% reduction in bacterial cell stiffness. With regard to cell adhesiveness, an increase was observed only following IVA treatment, by 7% relative to control cells. CSA-44 alone reduced adhesiveness by 32%, whereas treatment with IVA decreased it by 39%.

**Figure 6:**
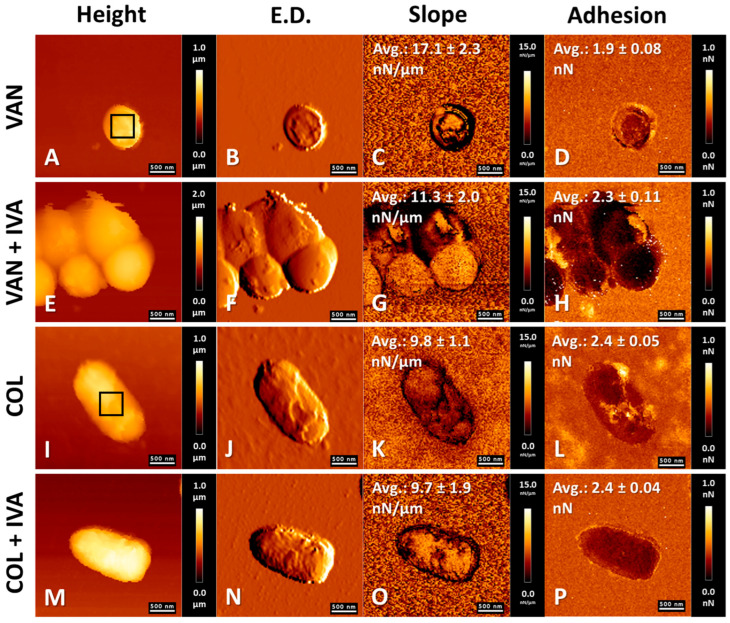
Effects of vancomycin (VAN) (1 µg/mL)/ivacaftor (IVA) (5 µg/mL) on *S. aureus* cells and colistin (COL) (1 µg/mL)/IVA (5 µg/mL) on *P. aeruginosa* cells. Atomic force microscopy (AFM) was used to assess topographical and nanomechanical properties under various modes: Height (**A**,**E**,**I**,**M**), Edge Detection (**B**,**F**,**J**,**N**), Slope (**C**,**G**,**K**,**O**), and adhesion (**D**,**H**,**L**,**P**). Panels (**A**–**D**) represent *S. aureus* cells treated with vancomycin; panels (**E**–**H**) illustrate the effects of combined treatment with VAN and IVA. Panels (**I**–**L**) represent *P. aeruginosa* cells treated with COL; panels (**M**–**P**) illustrate the effects of combined treatment with COL and IVA. A representative region used for nanomechanical parameter calculations is marked within panels (**A**,**I**). The calculated mean values ± SD are displayed within the respective panels. For each experimental condition, the values of the nanomechanical parameters were calculated from five separate bacteria cells.

The effects of the antibacterial agents administered alone and in combination with IVA on *S. aureus* and *P. aeruginosa* are presented in Figure 6. For Gram-positive cells, VAN induced morphological alterations (Figure 6B) and nanomechanical changes characterized by decreased stiffness and increased adhesion (Figure 6C,D). However, the inhibition of cell wall synthesis did not generally produce pronounced morphological alterations of *S. aureus* visible by AFM. The combination of VAN and IVA induced characteristic aggregation of *S. aureus* cells with concomitant surface alterations (Figure 6F), decreased stiffness (Figure 6G), and increased adhesion (Figure 6H). In Gram-negative cells, incubation with COL caused disruption of the outer membrane, manifested by surface alterations (Figure 6J) and decreased stiffness (Figure 6K) relative to untreated cells. The addition of IVA to COL did not induce aggregation of *P. aeruginosa*. Morphological changes induced by COL-IVA treatment were similar to those caused by COL alone (Figure 6N), with no additional differences in nanomechanical properties. VAN induced a 10.5% reduction in bacterial cell stiffness, whereas its combination with IVA resulted in an almost 41% decrease. At the same time, cell adhesiveness increased by 30% following VAN treatment and by 57.5% when combined with IVA. COL caused an approximately 27% reduction in bacterial cell stiffness. The addition of IVA did not further decrease this mechanical parameter. Regarding adhesiveness, both COL alone and the COL–IVA combination led to a 14% reduction relative to untreated bacteria.

### 3.6. Impact of Ceragenins CSA-13, CSA-44, and CSA-131 Combined with IVA on the Rheological Properties of P. aeruginosa (ATCC 27853) Biofilm

**Figure 7:**
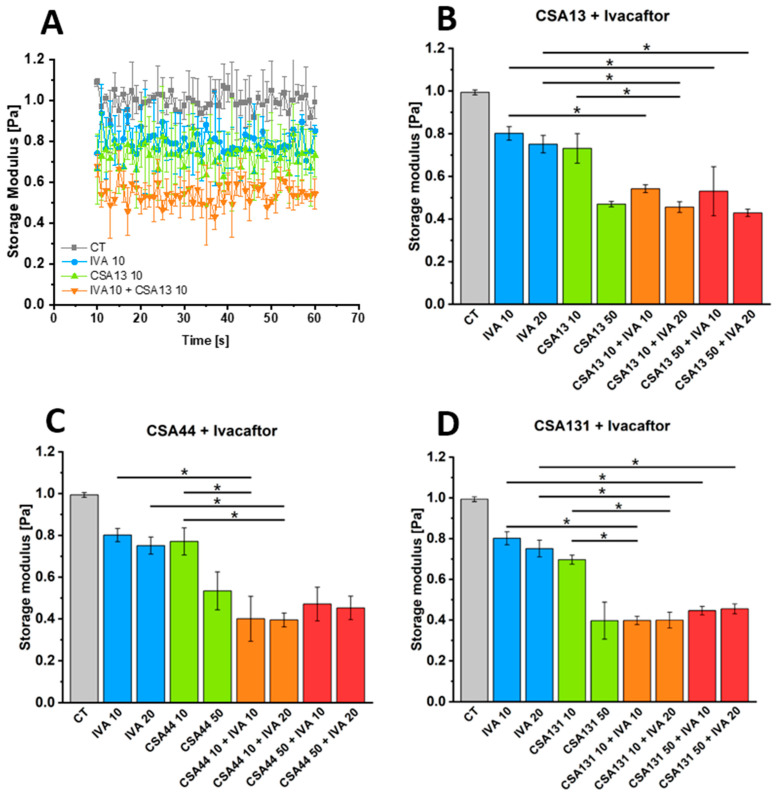
Storage modulus G’ of *P. aeruginosa biofilms* upon exposure to ceragenins CSA-13, CSA-44, and CSA-131 (at doses of 10 and 50 µg/mL) alone or when combined with ivacaftor (IVA, at doses of 10 and 20 µg/mL). Panel (**A**) demonstrates the storage modulus as a function of time, panels (**B**–**D**) demonstrate the average values of the storage modulus recorded for each sample during a 60 s analysis. Data are presented as mean ± SE. * indicates statistical significance when compared to single treatments.

To investigate the effect of ceragenins, IVA, and their combinations on the stiffness of *P. aeruginosa* biofilm, a shear rheometer was employed. Figure 7 presents the storage modulus (G’) values of the biofilms before and after the addition of ceragenin and IVA, either alone or in combination. The storage modulus of the control biofilm was around 1.0 Pa. For CSA-13, biofilm stiffness decreased by approximately 30% compared to the control. The addition of IVA further enhanced this effect, leading to a 40–50% reduction in G’ values. Treatment with CSA-44 alone resulted in about a 35% decrease in the storage modulus. When combined with IVA, the effect was more pronounced, reaching a 55–60% reduction. CSA-131 exhibited the strongest anti-biofilm activity among the tested ceragenins. When applied alone, CSA-131 decreased G’ values by about 45%, while the combination with IVA led to a nearly 65% reduction compared to control. IVA alone resulted in a stiffness decrease of approximately 10–15% for 10 µg/mL and up to 20% for a 20 µg/mL concentration compared to the control. When comparing the tested agents, CSA-131 was the most effective, followed by CSA-44, whereas CSA-13 showed the weakest activity. In all cases, the addition of IVA potentiated the anti-biofilm effect of ceragenins, resulting in a further significant decrease in biofilm stiffness. Those results are in agreement with a previous study indicating the ability of ceragenin to fluidize *P. aeruginosa* biofilm [51].

### 3.7. Assessment of Biocompatibility of Tested Agents

To assess the potential toxic effects of the selected compounds, three assays were employed: (i) hemolysis assay, in which the release of hemoglobin from damaged erythrocytes exposed to tested compounds is measured, (ii) MTT assay, measuring the viability of agent-exposed cells, and (iii) assessment of alterations in cell morphology upon addition of tested agents.

**Figure 8:**
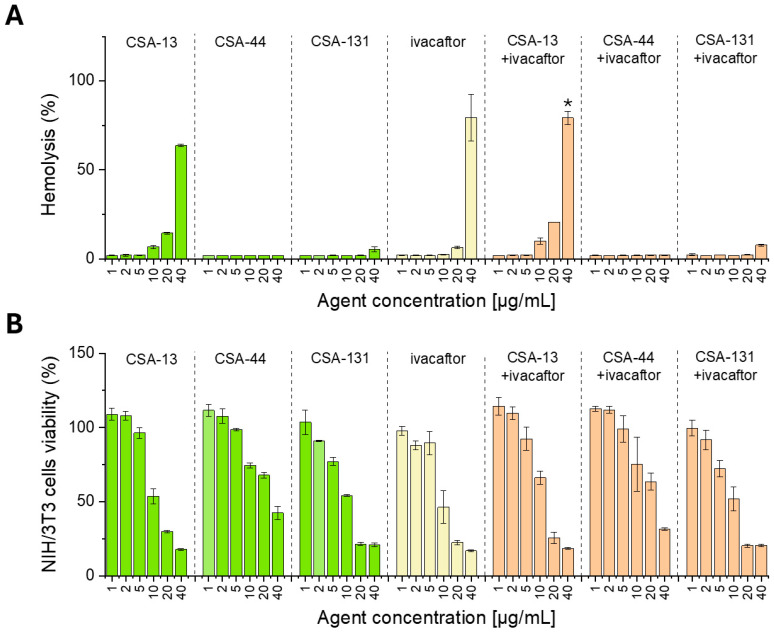
Hemo- and cytocompatibility of ceragenins CSA-13, CSA-44, and CSA-131 (green bars), ivacaftor (yellow bars), and ceragenins (all at a concentration range from 1 to 40 µg/mL) combined with ivacaftor (5 µg/mL) (orange bars). The release of hemoglobin from isolated erythrocytes and the viability of NIH/3T3 cells exposed to the tested agents are presented in panels (**A**,**B**), respectively. Results are presented as mean ± SD from three replicates. * indicates statistical significance when compared to single treatments.

As shown in Figure 8A, the tested compounds exhibit high hemocompatibility, as they do not induce significant hemoglobin release from erythrocytes at concentrations corresponding to their antimicrobial activity (up to 10 µg/mL). In most cases, the level of hemolysis remains low and does not exceed 3% in the range of 1–10 µg/mL. Only for CSA-13, ivacaftor, and combinations of these compounds, there is a significant increase in hemolysis, but only at much higher doses, i.e., 40 µg/mL.

At the same time, no significant cytotoxicity at antibacterial doses was observed, as confirmed by the MTT test, in which NIH/3T3 cells were exposed to the tested compounds for 24 h. At concentrations corresponding to their antimicrobial activity, cell survival remained above 70%, which indicates good biocompatibility of the compounds evaluated (Figure 8B).

**Figure 9:**
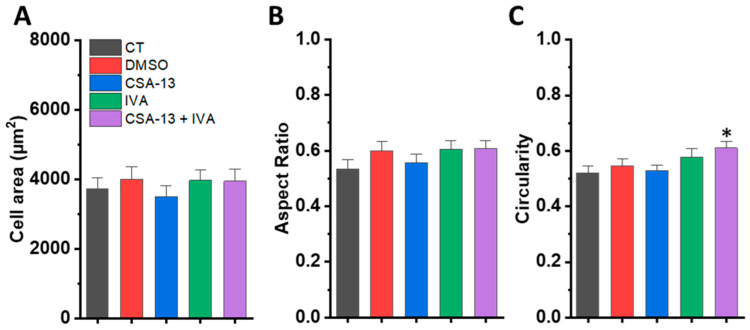
Quantitative analysis of fibroblast geometry treated with selected compounds. NIH 3T3 cells were treated with CSA-13 at a concentration of 5 µg/mL, IVA at a concentration of 5 µg/mL, and a combination of CSA-13 and IVA, both at 5 µg/mL. Controls included untreated cells cultured in cell medium and cells cultured in medium supplemented with 0.2% DMSO. The DMSO concentration corresponded to that used in the IVA-treated samples. Panel (**A**) illustrates the cell area measurements, panel (**B**) presents the aspect ratio analysis, and panel (**C**) depicts the cell circularity values. Data are presented as mean ± SE. * indicates statistical significance when compared to the untreated NIH 3T3 cells.

As additional confirmation of biocompatibility of tested formulations, assessment of NIH/3T3 cell morphology was performed, in which surface area, circularity, and the ratio of the smallest to the largest dimension of agent-exposed cells were estimated. The results of these measurements are shown in Figure 8. Cell surface area measurements indicated that the addition of CSA-13, IVA, DMSO alone, as well as combination treatment, did not significantly affect the size of the fibroblasts spread compared to the untreated cells (Figure 9A). No significant differences were also observed in the ratio of the smallest to the largest cell dimensions between untreated and treated cells (Figure 9B). Only increased circularity (Figure 9C) was observed in cells treated with CSA-13 and IVA together compared to untreated cells. The circularity of cells after combination treatment increased by 17% compared to untreated cells.

## 4. Discussion

Due to its worldwide occurrence, biofilm associated with CRS or CF chronic lung infection represents a significant medical problem for patients, physicians, and healthcare systems [1]. It is desirable to conduct new research to develop novel therapeutic options for these conditions. The purulent sputum causing obstruction in CF airways displays similarities to material accumulating in sinuses during CRS, posing a challenge to the efficacy of antibacterial molecules. For this reason, during the design of this current study, we decided to test the possibility of using IVA in combination with conventional antibiotics or ceragenins to suppress the planktonic bacterial cultures, such as *P. aeruginosa*, often causing CRS. *P. aeruginosa* deserves special attention because of the serious clinical challenges it poses. This is related to its multidrug resistance, which can be multifactorial and may be acquired through innate, acquired, or adaptive mechanisms. Additionally, *P. aeruginosa* may occur in the form of persistent cells, which further complicates eradication [52,53]. Previous studies indicate that the CYP107S1 enzyme, derived from *P. aeruginosa*, can metabolize multiple drugs from different classes. Results demonstrate binding and metabolism of the recombinant CYP107S1 enzyme of ciprofloxacin and fleroxacin, IVA, and a selective estrogen receptor-modulating antimicrobial adjuvant (raloxifene) [54]. Due to the co-occurrence of CRS and CF, IVA should remain of interest to scientists. Additionally, IVA therapy in patients with CF leads to a reduction in the features of CRS described on magnetic resonance imaging [55]. IVA, beyond its basic function, which is promoting the opening of the chloride channel created by the CFTR protein through phosphorylation, has an anti-biofilm effect through the inhibition of bacterial DNA gyrase and topoisomerase IV [41,42,56]. What is worth noting is that IVA does not negatively affect commonly used antibiotics [57]. CFTR modulators demonstrate measurable liquefaction/reduction in sputum viscoelasticity, which is associated with improved mucus clearance, enhanced lung function, and improved quality of life [58]. IVA action may also be effective on the pathological mucus present in CRS. On the other hand, we are aware that the benefits associated with IVA (increasing the penetration of pathological mucus by other drugs) are absent in CRS. Consequently, the effect of IVA in CRS may be related to the direct chemical properties of the drug. The standard IVA dose is 150 mg every 12 h. Pharmacokinetic information indicates that after oral administration, the maximum plasma concentration is reached after approximately 3–4 h and averages around 0.77 µg/mL [59]. Data on distribution into the respiratory tract are limited, but pharmacokinetic assessments have demonstrated that IVA concentrations in epithelial lining fluid (ELF) are substantially higher than in plasma. In an Australian regulatory report, ELF concentrations measured 4 h post-dose were approximately 8.1 times higher than concurrent plasma levels, corresponding to estimated values of around 6.2 µg/mL [60]. Currently, there are no registered indications for IVA other than CF. However, research is underway on the use of IVA in other diseases (CF-related and unrelated), such as pancreatitis, asthma, chronic obstructive pulmonary disease, and kidney and liver diseases [61,62,63,64]. The overall survival rate of CF patients receiving appropriate treatment (CFTR modulators, including ivacaftor) has improved significantly and has exceeded 60 years over the years. This demonstrates the high effectiveness of these medications in effectively clearing the airways and suggests their use in other conditions associated with mucus retention, such as CRS [65,66]. In our study, IVA (in doses similar to those found in the plasma during chronic IVA treatment in CF patients) demonstrated a synergistic effect with ceragenin CSA-13 and colistin. Due to its anti-biofilm activity and synergistic effect with tested agents, IVA may be an alternative in the treatment of resistant pathogens.

Our study, compared to other previously published studies, stands out for its innovative use of tested ceragenins, ivacaftor, and their combinations against bacteria associated with biofilm in chronic rhinosinusitis. The use of such drugs and their combinations have not been previously tested in the treatment of chronic rhinosinusitis. In this report, we reveal a complex relationship of activity and interaction, highlighting the potential of ceragenins as novel therapeutic agents. Previous studies demonstrated that tested ceragenins exhibit promising antibacterial and anti-biofilm activity against bacterial strains such as *S. aureus*, *S. pneumoniae*, *H. influenzae*, *M. catarrhalis,* and *P. aeruginosa*, which develop biofilm in CRS [67]. Importantly, and in accordance with previously reported findings, the present results further confirmed that ceragenins, i.e., CSA-13 and CSA-131, exert a pronounced impact on the metabolism and viability of *P. aeruginosa* Xen 5 and *S. aureus* Xen 30 compared to conventional drugs, i.e., COL and VAN. Niemirowicz et al. reported a statistically significant decrease in chemiluminescence intensity upon treatment with CSA-13 and CSA-131, whereas the reduction in light signal following exposure to conventional antibiotics was limited to approximately 20% [68,69].

The results reported herein show the potential for very broad and innovative use of ceragenins in medicine, which indicate that they should be of high interest to many medical specialties. Ceragenins can be combined with other compounds to enhance their activity (when reaching synergistic or additive effects), particularly with metallic nanoparticles. When presented to their targets on metallic nanoparticle surfaces in a core–shell nanosystem, they exhibit lower toxicity and enhanced and synergistic activity in combating planktonic bacteria, those organized in biofilms, and multidrug-resistant strains. Gold nanoparticles conjugated with ceragenins provide an alternative to standard treatments for otitis media and associated biofilm [70,71]. Ceragenins, such as CSA-131 in hydrogel form or others, are being studied and used to coat medical devices to prevent their colonization by microorganisms [72,73]. Ceragenins were also tested for orthopedics in connection with osteomyelitis and bacterial inflammation associated with implants [74].

AFM studies demonstrated the bactericidal properties of ceragenin CSA-44 against *S. aureus* and *P. aeruginosa* cells. Alterations were observed on the bacterial cell surfaces, as well as micro-leakages from the cell interior. The physicochemical properties of the cell surfaces were also changed. Results are consistent with those reported in previous reports, where the effects of selected ceragenins on both Gram-positive and Gram-negative bacteria were investigated [75,76,77]. The activity of the antibiotics COL and VAN against the Gram-positive and Gram-negative bacterial cells was also confirmed, in agreement with reported findings [78,79]. The added value in this study is that the effects of IVA, both alone and in combination with CSA-44 and selected antibiotics, on the morphology and mechanical properties of *S. aureus* and *P. aeruginosa* cells were demonstrated. A specific effect of IVA on Gram-positive bacterial cells was observed, resulting in bacterial aggregate formation. A synergistic effect of IVA in combination with ceragenin CSA-44 and VAN on bacterial cells was also demonstrated. The state only applies to results observed in AFM studies.

Rheological analyses demonstrated that ceragenin CSA-13 reduces the stiffness of *P. aeruginosa* biofilms. An addition of IVA seems to cause a further minor reduction in stiffness, although this effect is subtle. This difference could indicate a synergistic effect, meaning the combined treatment is more effective than CSA-13 alone at altering bacterial cell wall properties. Mechanical changes in biofilm that disrupt its structure make it more exposed to the action of phagocytes and easier to eradicate [80]. This study reveals that the compression-stiffening and shear-softening behavior of *P. aeruginosa*, *S. aureus*, and *C. albicans* biofilms can be replicated in entangled DNA networks with added microbial cells, and that these effects are cell-type dependent. Furthermore, cations (Mg^2+^, Ca^2+^, and Cu^2+^) and *P. aeruginosa* native bacteriophage Pf1 significantly impact biofilm viscoelasticity [81]. Another study reveals that functional amyloid fibrils produced by the *fap* operon significantly enhance the mechanical strength of *P. aeruginosa* biofilms by increasing hydrophobicity and stiffness. Disruption of the *fap* operon eliminates this stiffening, demonstrating the amyloid’s critical role in biofilm integrity [82]. These findings underline the critical role of DNA and highlight amyloidogenic pathways as a promising target for novel anti-biofilm strategies against persistent *P. aeruginosa* infections.

However, this study had some limitations, as it was conducted in vitro, and the results require confirmation through in vivo studies. A limited number of bacterial strains was also investigated. Considering a broader spectrum of strains, including those with various antibiotic resistance profiles, could provide a more comprehensive picture of the efficacy of the tested anti-biofilm agents. Further in vivo studies are necessary to confirm the in vitro findings and evaluate the safety of ceragenins in treating CRS. Investigating potential interactions between ceragenins and other components of the immune system should also be considered. In summary, this research provides compelling evidence for the promising effect of ceragenins in combating bacterial biofilms in CRS. Synergistic action with IVA could represent a breakthrough in treating these persistent infections. However, further research is needed to fully assess the therapeutic potential of ceragenins and optimize treatment strategies.

## Figures and Tables

**Table 1:** Minimal inhibitory concentrations (MICs) and minimal bactericidal concentrations (MBCs) of ceragenins (CSA-13, CSA-44, and CSA-131), ivacaftor (IVA), and conventional antibiotics (vancomycin [VAN], colistin [COL], meropenem [MEM]) against bacteria causing upper respiratory tract or sinus infection—reference and clinical strains. (n/d—not determined).

Strain	MIC/MBC [µg/mL]						
	CSA-13	CSA-44	CSA-131	IVA	VAN	COL	MEM
*Staphylococcus aureus* (ATCC 29213)	0.5/2	1/8	1/8	2/64	2/16	n/d	n/d
*Staphylococcus aureus* MRSA (clinical strain 1)	<0.5/<0.5	1/2	1/2	4/16	<0.5/2	n/d	n/d
*Staphylococcus aureus* MRSA (clinical strain 2)	<0.5/2	1/4	1/4	4/4	<0.5/2	n/d	n/d
*Staphylococcus aureus* MRSA (clinical strain 3)	<0.5/<0.5	1/4	1/1	8/16	1/2	n/d	n/d
*Staphylococcus aureus* MRSA (clinical strain 4)	<0.5/<0.5	1/1	1/1	4/16	<0.5/1	n/d	n/d
*Staphylococcus aureus* MRSA (clinical strain 5)	<0.5/<0.5	1/2	1/1	2/4	<0.5/<0.5	n/d	n/d
*Staphylococcus aureus* MRSA (clinical strain 6)	<0.5/<0.5	1/4	1/4	2/8	<0.5/1	n/d	n/d
*Staphylococcus aureus* MRSA (clinical strain 7)	1/1	1/1	1/2	16/16	1/1	n/d	n/d
*Staphylococcus aureus* MSSA (clinical strain 1)	4/4	2/2	1/1	8/16	1/2	n/d	n/d
*Staphylococcus aureus* MSSA (clinical strain 2)	<0.5/1	2/2	1/2	16/32	1/2	n/d	n/d
*Streptococcus pneumoniae* (ATCC 49619)	<0.5/<0.5	1/1	0.5/1	1/1	<0.5/1	n/d	n/d
*Streptococcus pneumoniae* (clinical strain 2)	<0.5/<0.5	<0.5/<0.5	<0.5/<0.5	<0.5/1	<0.5/<0.5	n/d	n/d
*Streptococcus pneumoniae* (clinical strain 3)	2/8	2/4	2/2	8/16	1/1	n/d	n/d
*Streptococcus pneumoniae* (clinical strain 4)	<0.5/<0.5	<0.5/<0.5	0.5/0.5	1/1	0.5/0.5	n/d	n/d
*Streptococcus pneumoniae* (clinical strain 5)	<0.5/<0.5	<0.5/<0.5	<0.5/<0.5	<0.5/1	<0.5/1	n/d	n/d
*Streptococcus pneumoniae* (clinical strain 6)	<0.5/<0.5	1/1	<0.5/<0.5	1/1	<0.5/1	n/d	n/d
*Streptococcus pneumoniae* (clinical strain 7)	<0.5/1	1/1	<0.5/<0.5	1/2	<0.5/1	n/d	n/d
*Streptococcus pneumoniae* (clinical strain 8)	<0.5/1	0.5/2	<0.5/<0.5	1/2	<0.5/<0.5	n/d	n/d
*Streptococcus pneumoniae* (clinical strain 9)	<0.5/<0.5	<0.5/1	<0.5/<0.5	<0.5/1	<0.5/<0.5	n/d	n/d
*Streptococcus pneumoniae* (clinical strain 10)	<0.5/1	1/1	<0.5/<0.5	1/2	<0.5/1	n/d	n/d
*Streptococcus intermedius* (clinical strain 1)	0.5/0.5	1/1	0.25/1	1/1	1/2	n/d	n/d
*Streptococcus anginosus* (clinical strain 1)	<0.5/<0.5	1/1	<0.5/0.5	2/2	1/1	n/d	n/d
*Streptococcus pyogenes* (clinical strain 1)	<0.5/<0.5	<0.5/<0.5	<0.5/<0.5	1/2	<0.5/<0.5	n/d	n/d
*Moraxella catarrhalis* (ATCC 25238)	0.5/2	0.5/1	<0.5/1	0.5/0.5	n/d	n/d	<0.5/1
*Haemophilus influenzae* (ATCC 49766)	<0.5/1	<0.5/0.5	<0.5/0.5	<0.5/<0.5	n/d	n/d	<0.5/<0.5
*Pseudomonas aeruginosa* (ATCC 27853)	2/4	2/2	1/1	>128/>128	n/d	<0.5/<0.5	n/d
*Pseudomonas aeruginosa* (clinical strain 1)	2/<4	2/2	1/1	>256/>256	n/d	<0.5/<0.5	n/d
*Pseudomonas aeruginosa* (clinical strain 2)	<0.5/<0.5	2/2	4/8	32/>64	n/d	<0.5/<0.5	n/d
*Pseudomonas aeruginosa* (clinical strain 3)	4/8	2/4	2/4	>256/>256	n/d	<0.5/<0.5	n/d
*Pseudomonas aeruginosa* (clinical strain 4)	<0.5/<0.5	1/1	<0.5/<0.5	16/16	n/d	<0.5/<0.5	n/d
*Pseudomonas aeruginosa* (clinical strain 5)	<0.5/1	1/2	1/1	>256/>256	n/d	<0.5/1	n/d
*Pseudomonas putida* (clinical strain 1)	0.5/1	1/2	1/2	64/>128	n/d	<0.5/<0.5	n/d

**Table 2:** Fractional inhibitory concentration index (FICI) calculated for ceragenins/antibiotics combined with ivacaftor (IVA), vancomycin (VAN), colistin (COL), and meropenem (MEM) against sinus infection-causing bacteria. If the MIC for the compound was lower or higher than the concentration range tested, the limiting concentrations were taken for the calculations, and those combinations are denoted by *. FICI < 0.5 indicates synergy (S), 0.5–4 indifference (I), and >4 antagonism (A). (n/d—not determined).

Strain	FICI of Antimicrobials/IVA Combinations					
	CSA-13	CSA-44	CSA-131	VAN	COL	MEM
*Staphylococcus aureus* (ATCC 29213)	0.625 (I)	0.75 (I)	1.5 (I)	1.25 (I)	n/d	n/d
*Streptococcus pneumoniae* (ATCC 49619)	1.25 (I)	8 (A)	1.5 (I)	1.125 (I)	n/d	n/d
Haemophilus influenzae (ATCC 49766)	2 (I) *	2 (I) *	2 (I) *	n/d	n/d	2 (I) *
*Moraxella catarrhalis* (ATCC 25238)	1 (I) *	1 (I)	1.5 (I) *	n/d	n/d	1.5 (I) *
*Pseudomonas aeruginosa* (ATCC 27853)	0.265 (S) *	1.02 (I) *	8.125 (A) *	n/d	0.128 (S) *	n/d
**Strain**	**FICI of Ceragenins/VAN Combinations**					
	**CSA-13**		**CSA-44**		**CSA-131**	
*Staphylococcus aureus* (ATCC 29213)	1.0625 (I)		2.25 (I)		0.5625 (I)	
*Streptococcus pneumoniae* (ATCC 49619)	1.03125 (I)		0.5625 (I)		1.0625 (I)	
**Strain**	**FICI of Ceragenins/COL Combinations**					
	**CSA-13**		**CSA-44**		**CSA-131**	
*Pseudomonas aeruginosa* (ATCC 27853)	0.1875 (S)		0.75 (I)		0.75 (I)	
**Strain**	**FICI of Ceragenins/MEM Combinations**					
	**CSA-13**		**CSA-44**		**CSA-131**	
*Haemophilus influenzae* (ATCC 49766)	2 (I) *		2 (I) *		2 (I) *	
*Moraxella catarrhalis* (ATCC 25238)	0.75 (I) *		0.75 (I) *		1.25 (I) *	

## Data Availability

The authors confirm that the data supporting the findings of this study are available at the reader’s request.

## Abbreviations

The following abbreviations are used in this manuscript:

CRS	Chronic rhinosinusitis
CF	Cystic fibrosis
CFTR	Cystic fibrosis transmembrane regulator
CSAs	Ceragenins
IVA	Ivacaftor
CSA	Ceragenin
MIC	Minimal inhibitory concentration
MBC	Minimal bactericidal concentration
FICI	Fractional inhibitory concertation index
AFM	Atomic force microscopy
RS	Rhinosinusitis
ARS	Acute rhinosinusitis
DNA	Deoxyribonucleic acid
VAN	Vancomycin
COL	Colistin
MEM	Meropenem
QI	Quantitative imaging
ELF	Epithelial lining fluid
